# Feeding an unsalable carrot total-mixed ration altered bacterial amino acid degradation in the rumen of lambs

**DOI:** 10.1038/s41598-023-34181-0

**Published:** 2023-04-28

**Authors:** Daniel L. Forwood, David J. Innes, Mariano C. Parra, Terra Stark, David P. de Souza, Alex V. Chaves, Sarah J. Meale

**Affiliations:** 1 School of Agriculture and Food Sciences, Faculty of Science, The University of Queensland, Gatton, Australia; 2 Australian Institute for Bioengineering and Nanotechnology, The University of Queensland, St Lucia, Australia; 3 Metabolomics Australia, Bio21 Institute, The University of Melbourne, Parkville, Australia; 4 School of Life and Environmental Sciences, Faculty of Science, The University of Sydney, Camperdown, Australia

**Keywords:** Applied microbiology, Microbiome, Metabolomics

## Abstract

The objective of this study was to determine the influence of a total-mixed ration including unsalable carrots at 45% DM on the rumen microbiome; and the plasma, rumen and liver metabolomes. Carrots discarded at processing were investigated as an energy-dense substitute for barley grain in a conventional feedlot diet, and improved feed conversion efficiency by 25%. Here, rumen fluid was collected from 34 Merino lambs at slaughter (n = 16 control; n = 18 carrot) after a feeding period of 11-weeks. The V4 region of the 16S rRNA gene was sequenced to profile archaeal and bacterial microbe communities. Further, a comprehensive, targeted profile of known metabolites was constructed for blood plasma, rumen fluid and biopsied liver metabolites using a gas chromatography mass spectrometry (GC–MS) metabolomics approach. An in vitro batch culture was used to characterise ruminal fermentation including gas and methane (CH_4_) production. In vivo rumen microbial community structure of carrot fed lambs was dissimilar (*P* < 0.01; PERMANOVA), and all measures of alpha diversity were greater (*P* < 0.01), compared to those fed the control diet. Unclassified genera in *Bacteroidales* (15.9 ± 6.74% relative abundance; RA) were more abundant (*P* < 0.01) in the rumen fluid of carrot-fed lambs, while unclassified taxa in the *Succinivibrionaceae* family (11.1 ± 3.85% RA) were greater (*P* < 0.01) in the control. The carrot diet improved in vitro ruminal fermentation evidenced as an 8% increase (*P* < 0.01) in DM digestibility and a 13.8% reduction (*P* = 0.01) in CH_4_ on a mg/ g DM basis, while the control diet increased (*P* = 0.04) percentage of propionate within total VFA by 20%. Fourteen rumen fluid metabolites and 27 liver metabolites were influenced (*P* ≤ 0.05) by diet, while no effect (*P* ≥ 0.05) was observed in plasma metabolites. The carrot diet enriched (impact value = 0.13; *P* = 0.01) the tyrosine metabolism pathway (acetoacetic acid, dopamine and pyruvate), while the control diet enriched (impact value = 0.42; *P* ≤ 0.02) starch and sucrose metabolism (trehalose and glucose) in rumen fluid. This study demonstrated that feeding 45% DM unsalable carrots diversified bacterial communities in the rumen. These dietary changes influenced pathways of tyrosine degradation, such that previous improvements in feed conversion efficiency in lambs could be explained.

## Introduction

Ruminants are well-known for their unique ability to convert inedible plant and plant-derived materials into high quality, human-edible products. Degradation of these feedstuffs occurs predominantly in the rumen, where a consortium of microbiota produces metabolites that are utilised for microbial growth, whole-animal maintenance, muscle, milk and fibre production^1,2^. Metabolites have been quantified in the rumen^2^ and are important markers in the identification of cell processes that underpin and influence the function of an organism^3^. The relationship between the rumen microbiome and the host has mainly been explored in animals fed conventional maize grain diets^4^. Metabolites including histamine, putrescine and tyramine were correlated with *Acetitomaculum* and *Butyrivibrio* in the rumen of maize grain-fed animals^4^, acting as markers for rumen acidosis^5^. Other markers, including metabolites present in blood plasma, provide information on performance and yield parameters including growth rate or carcass weight^6^, while liver metabolites best indicate the interaction of diet on nutrient utilisation^7^. To our knowledge, no other studies on the influence of alternative feedstuffs on the plasma, hepatic or rumen fluid metabolites using an ovine model have been reported in the literature.

In Australia, vegetable waste comprises approximately 29% of total food wastage^8^, but its use as livestock feed is seldom employed as an avenue of waste mitigation. Feeding a range of vegetables including beans, cabbage, cauliflower and potato maintained dry matter (DM) intake, average daily gain and DM digestibility in 12–18-month-old *Bos indicus* bulls at up to 9.7% DM of a wheat bran-based diet^9^, while the liveweight and average daily gain of Rambouillett × Polypay wethers fed a mixed ration substituting 50% DM sorghum grain for 50% DM onion bulbs was also maintained at 0.14 kg/day^10^. Vegetables have also demonstrated promise, where feeding whole potatoes at 30% DM of a finishing diet increased final liveweight, and dressing percentage by 5.4 and 2.7% respectively in double-muscled cows^11^, while carrots fed at 45% DM of a total-mixed ration (TMR) improved feed conversion efficiency in Merino wethers by 25% compared to a conventional grain-based TMR^12^. Diets containing feedstuffs high in fermentable sugars and readily degradable carbohydrates, such as carrots^13^ provide an environment rich in substrates readily utilised by rumen microbes, improving microbial activity^14^ and ruminant performance by extension.

Therefore, the aim of this study was to determine the influence of feeding lambs a TMR including 45% (DM-basis) unsalable carrots on the microbial community structure within the rumen and to profile the plasma, liver, and rumen fluid metabolomes to understand the systemic influence of diet. It was hypothesised that the carrot diet would provide an environment conducive to higher bacterial diversity, resulting in greater variation in the rumen metabolic profile compared to conventional feed ingredients (e.g. control diet).

## Materials and methods

### Animal experiment and sample collection

The animal study was previously detailed by Forwood et al.^12^. The methodology was performed in accordance with the guidelines of the Australian code for the care and use of animals for scientific purposes (2013), and was approved by The University of Queensland Animal Ethics Committee (approval #SAFS/035/19). The study is reported in accordance with the ARRIVE guidelines. Thirty-six 7-month-old Merino wether lambs, initial live weight 24.7 ± 0.3 kg were randomly allocated by live weight and assigned to individual indoor pens. After two-weeks adaptation, lambs were fed either control (51.9% barley grain, 40.2% lucerne hay, 7.1% canola meal, 0.8% MegaMin Mineral Blend); or carrot (45.2% carrot, 8.9% barley grain, 30.0% lucerne hay, 15.1% canola meal, 0.8% MegaMin Mineral Blend) experimental diets on a DM-basis for 11-weeks. Diets were formulated to be isonitrogenous. Twenty-four hours after completion of the feeding period, blood was obtained from lambs through jugular venepuncture into 10 mL lithium-heparin vacutainer tubes and placed on ice^15^. Samples were centrifuged at 2500×*g* for 15 min with resultant plasma stored at − 80 °C for metabolomics analysis.

Experimental details regarding the commercial slaughter of lambs have been reported^12^. Here, rumen digesta was collected immediately post-mortem from four locations within the rumen. Contents were pooled, then strained through two layers of muslin cloth, and 1 mL subsamples were immediately frozen in liquid nitrogen and stored at − 80 °C for DNA and metabolomics analysis.

### Volatile fatty acids (VFA)

Organic acids and VFA were determined in rumen fluid samples using previously described methods^16^. Samples were processed and analysed via an Agilent technologies 7820A gas–liquid chromatograph system set up according to described methods^17^. Total volatile fatty acid concentration were expressed in mM and individual VFA as percentages of total VFA. Predicted CH_4_ yield was predicted using the following equation^18^:

$${Predicted\, CH}_{4}\, yield=0.50A+0.25P+0.50B,$$

where A = acetate; P = propionate and B = butyrate.

### Rumen fluid DNA extraction, 16S rRNA sequencing, and diversity

Genomic DNA (gDNA) was extracted from rumen samples using bead-beating and on-column purification, per the methods used by Popova et al.^19^. DNA extracts were quantified on a Nanodrop 1000 Spectrophotometer (Thermo Fisher Scientific, France) and run on a FlashGel System (Lonza, Rockland, Inc.) to check integrity. Approximately 15 µg of gDNA were sent to Génome Québec (Montréal, QC H3B 1S6, Canada) for fluidigm amplification and MiSeq Illumina sequencing. The 515F, paired with 806R primers were selected for amplification targeting the V4 region of 16S rRNA gene of bacteria.

The DADA2 v. 1.8^20^ package was used in R v. 4.0.2^21^ within RStudio 1.3.959^22^ to process the 16S rRNA gene sequences. Briefly, forward and reverse 16S rRNA gene sequences were trimmed to 220 and 200 bp, respectively, merged, and then chimeras were removed. Taxonomy was assigned to the remaining sequences, referred to amplicon sequence variants (ASVs) at 100% similarity using the RDP naïve Bayesian classifier and the Greengenes database^23^. The number of ASVs per sample, Shannon diversity index, and inverse Simpson’s diversity index for 16S rRNA gene datasets were calculated in R using Phyloseq v. 1.26.0^24^. All 16S rRNA gene sequences were submitted to the NCBI Sequence Read Archive under BioProject accession number PRJNA772293.

### Comprehensive targeted metabolomics

Plasma for comprehensive, targeted metabolomics analyses were prepared following the methods of Zheng et al.^25^ with adjustments, where 100 µL of sample was combined with 300 µL methanol and vortexed for 15 s. Samples were centrifuged at 16,000×*g* for 10 min at 4 °C. Subsequently, an aliquot of 50 µL supernatant was placed into 1.5 mL Eppendorf tubes and dried under vacuum (Eppendorf Concentrator Plus, Hamburg, Germany). Rumen fluid was similarly treated, except 30 µL of each sample was pipetted into 1.5 mL Eppendorf tube and dried under vacuum.

Liver samples previously stored at − 80 °C were held on dry ice, then manually pulverised to obtain approximately 20–30 mg frozen liver and combined with 600 µL extraction solution [3:1 (v/v) methanol:water including 13C-sorbitol and 13C,15N-valine as internal standards]. Samples were homogenised via bead-beating on a Bead Ruptor Elite Bead Mill Homogenizer (Omni International, Kennesaw, GA, USA) for 5 cycles at 0 °C, each comprising 30 s bead beating at 4.00 m/s, followed by a dwell period of 45 s. Homogenate was mixed with 120 µL chloroform (chloroform:methanol:water = 1:3:1 v/v monophasic mixture), vortexed vigorously then incubated on ice for 10 min on an orbital shaker at medium speed. Samples were centrifuged at 0 °C for 5 min at maximum speed, and supernatant transferred for GC–MS preparation. Aliquots of 10 and 50 µL supernatant, along with a 200 µL pooled biological quality control were dried under vacuum. A final dry-down step was conducted using 50 µL methanol.

An aliquot of each sample (50 µL) was pooled to generate a pooled biological quality control sample (PBQC) and aliquots (50 µL) of this mixture were evaporated to dryness. Samples and PBQCs were derivatised by methoxyamination by the addition of 25 µL methoxyamine (30 mg/mL in pyridine, 2 h, 37 °C, 900 rpm), followed by trimethylsilylation with 25 µL BSTFA + 1% TMCS (1 h, 37 °C, 900 rpm). Metabolite profiles were acquired on a Shimadzu GCMS-TQ8050 NX system (Shimadzu, Kyoto, Japan).

Approximately 0.5 µL of derivatised sample was injected into the GC inlet set at 280 °C in split mode of 1:10. Chromatographic separation was achieved using an Agilent DB-5 capillary column (30 m × 0.25 mm × 1 µm). Oven conditions were set at 100 °C starting temperature, held for 4 min, then ramped at 10 °C/min to 320 °C and held for 11 min. Helium was used as the carrier gas at a flow rate of 1 mL/min. Compounds were fragmented by electron ionisation and analysed in multiple reaction monitoring (MRM) mode using the Shimadzu Smart Metabolites Database (https://www.shimadzu.com/an/gcms/metabolites/index.html) containing 475 MRM metabolite targets. A high-quality matrix was manually curated using the Shimadzu LabSolutions Insight GCMS program (v.3.7 SP3, Shimadzu Corporation), where metabolite targets were removed from the dataset if they were not present in all samples.

### In vitro incubation

In vitro incubations of the carrot and control diets (composition as fed to lambs) were conducted at The University of Queensland (Gatton, QLD). Three Holstein steers were used for these incubations under the approval of The University of Queensland Animal Ethics Committee (approval #AE35581), as donor species does not influence rumen microbial composition^26^. Rumen digesta was collected from each animal within four distinct regions of the rumen as previously described^27^. Digesta was pooled and liquor strained through four layers of cheesecloth into a pre-warmed 1 L thermos and immediately transported to the laboratory. The methods of Menke et al.^28^ were used for the preparation of rumen inoculum, while the methods of Forwood et al.^17^ were modified for the incubation. Briefly, bottles (n = 18) containing 25 mL inoculum solution (65% buffer; 32% rumen liquor; 3% reducing agent) and ANKOM bags containing 0.5 g DM of each treatment were incubated using an orbital shaker (Ratek Instruments, Boronia, Australia) heated to 39 °C and set to 120 rpm. Moreover, these analyses comprised three 24 h incubation runs, with three replicates of each of carrot and control diet per run.

Samples of gas and methane were collected at 6 h and 24 h incubation. Following the methods of Forwood et al.^17^ with adjustments, 18 mL gas samples were extracted and transferred into 12 mL evacuated Exetainers (Labco Ltd., High Wycombe, United Kingdom) for CH_4_ determination. The CH_4_ concentration was measured using an Agilent model 7890a gas chromatograph with a flame ionization detector (FID) calibrated to 250 °C, air flow 300 mL/min, H_2_ fuel flow 30 mL/min, makeup flow (N_2_) 30 mL/min installed with a capillary column (Restek Rt-Q-Bond, 30 m × 0.53 mmID × 20 μm). The Split-Splitless Inlet was heated to 60 °C, 9.526 PSI, with Helium total flow 33 mL/min, septum purge flow 3 mL/min, Split ration 5:1, Split Flow 25 mL/min and an oven temperature of 60 °C. Methane measurements were defined as mg CH_4_/g DM and mg CH_4_/g digested DM.

Total gas production was obtained immediately after CH_4_ sampling using a water displacement apparatus^29^, while pH of the rumen fluid was measured using a Hanna Edge HI2002 pH meter (Hanna Instruments, Woonsocket, RI, USA). After gas and pH samples were collected, bottles were placed on ice to prevent further fermentation. Bags were removed from the bottles and rinsed thoroughly with water for 1 h in a washing machine, followed by drying in an oven set to 65 °C until a constant weight. In vitro dry matter disappearance was estimated by weighing the dry bags.

### Statistical analyses

Alpha diversity and volatile fatty acids were analysed using the MIXED procedure of SAS 9.4 (SAS Institute, Cary NC), with treatment as the fixed effect and sample within treatment as the random effect. In vitro, gas production and methane data were similarly analysed, except for the addition of incubation run and treatment within run as random effects. The effect of diet on the microbial community structures was determined using PERMANOVA (adonis2 function) and Bray–Curtis dis-similarities in R with vegan 2.5-6^30^. Statistical significance for all data analysed via the MIXED procedure was declared if the *P*-value was < 0.05, and a tendency reported if 0.05 ≤ *P* ≤ 0.10. Further, significance for log2 fold change data for archaeal and bacterial abundance was declared if false discovery (FDR) adjusted *P*-value ≤ 0.05.

Univariate and multivariate statistical analyses were conducted on plasma, rumen and liver metabolome data using Metaboanalyst 5.0 (https://www.metaboanalyst.ca). Data were normalised to the median and log-transformed, then pareto-scaled to reduce variability and the likelihood of false discovery. Fold change analyses were conducted at set thresholds of fold change = 1.2 and FDR *P*-value = 0.05 to determine significantly different metabolites and reduce the potential for false positive readings. Once significantly expressed metabolites were identified, pathway analysis was conducted using Metaboanalyst 5.0 based on the *Bos taurus* library and Kyoto encyclopaedia of genes and genomes (KEGG) pathway database to assign metabolites to metabolic pathways^31^. Principal component analyses (PCA) were used to determine separation of differentially expressed metabolites by treatment effect, while orthogonal projections to latent structures discriminant analysis (OPLS-DA) was utilised to ascertain strength of separation. To determine validity of the OPLS-DA model, n = 100 permutation tests were run on the data, where biological significance, thus robustness of the model was declared with Q^2^ and R^2^ values > 0.9^32^.

To elucidate the metabolic contribution of rumen microbes to systemic function, functional profiles of rumen microbes were predicted using the CowPI platform (https://www.cowpi.org/^33^). Subsequently, the influence of functional pathway on systemic differences between carrot and control-fed lambs was ascertained through the linear discriminant analysis effect size (LEfSe; https://huttenhower.sph.harvard.edu/galaxy/^34^) tool, through the Galaxy platform (version 1.39.5.0 (https://galaxyproject.org/^35^). Linear discriminant analyses scores were declared significantly different if scores were ≥ 2.0 and *P* ≤ 0.05^36^.

The interaction between bacterial and archaeal genera, and metabolites in rumen fluid were analysed by a Spearman’s rank correlation using the “psych” package on R software (v. 4.0.2^21^;^37^), and visualised using a heat map generated by the packages “ggcorrplot”^38^ and “corrplot” (v. 0.90^39^). Pathways from functional analyses derived from differentially expressed rumen microbes were similarly visualised.

### Ethics approval and consent to participate

Rumen fluid and liver samples used in this study were obtained from lambs in a companion study under the approval of The University of Queensland Animal Ethics Committee SAFS/035/19.

## Results

### Rumen fermentation

#### In vitro fermentation characteristics, gas and CH_4_ production

In vitro gas production (mL/g DM) was similar between control and carrot diets (*P* = 0.28; Table 1) despite an 8.8% increase (*P* < 0.01) in in vitro dry matter digestibility (IVDMD) in carrot compared to control diet. Methane, when expressed as CH_4_ mg per g DM incubated was 13.8% lower for the carrot diet (*P* = 0.01) and tended (*P* = 0.10) to be lower when expressed as a percentage of total gas, compared to the control.

**Table 1:** Volatile fatty acid (VFA) profile and pH of rumen fluid obtained post-slaughter from lambs fed a control or carrot diet.

	Control	Carrot	SEM	*P*-value
In vivo				
Rumen pH	5.9	6.4	0.12	< 0.01
Total VFA, mM	111.8	99.4	8.53	0.31
Predicted CH_4_ yield, mol/100 mol TVFA^1^	40.7	39.4	0.32	0.01
Percentages of individual VFA, %				
Acetate (A)	52.6	52.7	0.7	0.94
Propionate (P)	22.9	19	1.26	0.04
Butyrate (B)	17.3	16.6	0.95	0.6
BCVFA	4.13	8.3	0.42	< 0.01
Valerate	2.43	2.92	0.2	0.09
Caproate	0.64	0.56	0.11	0.61
A:P ratio	2.51	2.84	0.15	0.13
In vitro				
Fermentation characteristics				
pH	6.68	6.77	0.06	< 0.01
Gas production, mL/g DM	128	124	2.55	0.28
CH_4_, %	18.9	16.9	0.68	0.1
CH_4_, mg/g DM	17.4	15	0.72	0.01
IVDMD, %	55.8	60.7	0.84	< 0.01

In vitro fermentation characteristics, CH_4_ production and dry matter digestibility of diets determined using rumen fluid obtained from cannulated Holstein steers.

*SEM* standard error of the mean, *IVDMD* in vitro dry matter digestibility, *BCVFA* branch-chained volatile fatty acids (iso-valerate + iso-butyrate).

^1^CH_4_ yield = 0.50A + 0.25P + 0.50B (Williams et al.^18^).

#### Rumen pH, VFA and predicted CH_4_ yield from lambs

The pH of rumen fluid collected immediately post-slaughter was higher (*P* < 0.01; Table 1) for carrot-fed lambs. There was no effect (*P* ≥ 0.13) of diet on the total concentration of VFA, or individual percentage of acetate, butyrate, caproate acids, or the A:P in rumen fluid. As a proportion of total VFA, branch-chained volatile fatty acids (BCVFA) were twofold greater (*P* < 0.01) and valerate tended (*P* = 0.09) to be greater with the carrot diet, whereas, propionate was greater (*P* = 0.04) in rumen fluid of control lambs. Further, predicted CH_4_ yield (mol/100 mol TVFA) decreased (*P* = 0.01) by 3.2% in carrot-fed lambs, compared to control-fed lambs.

### Rumen archaeal and bacterial community structure and diversity

**Figure 1:**
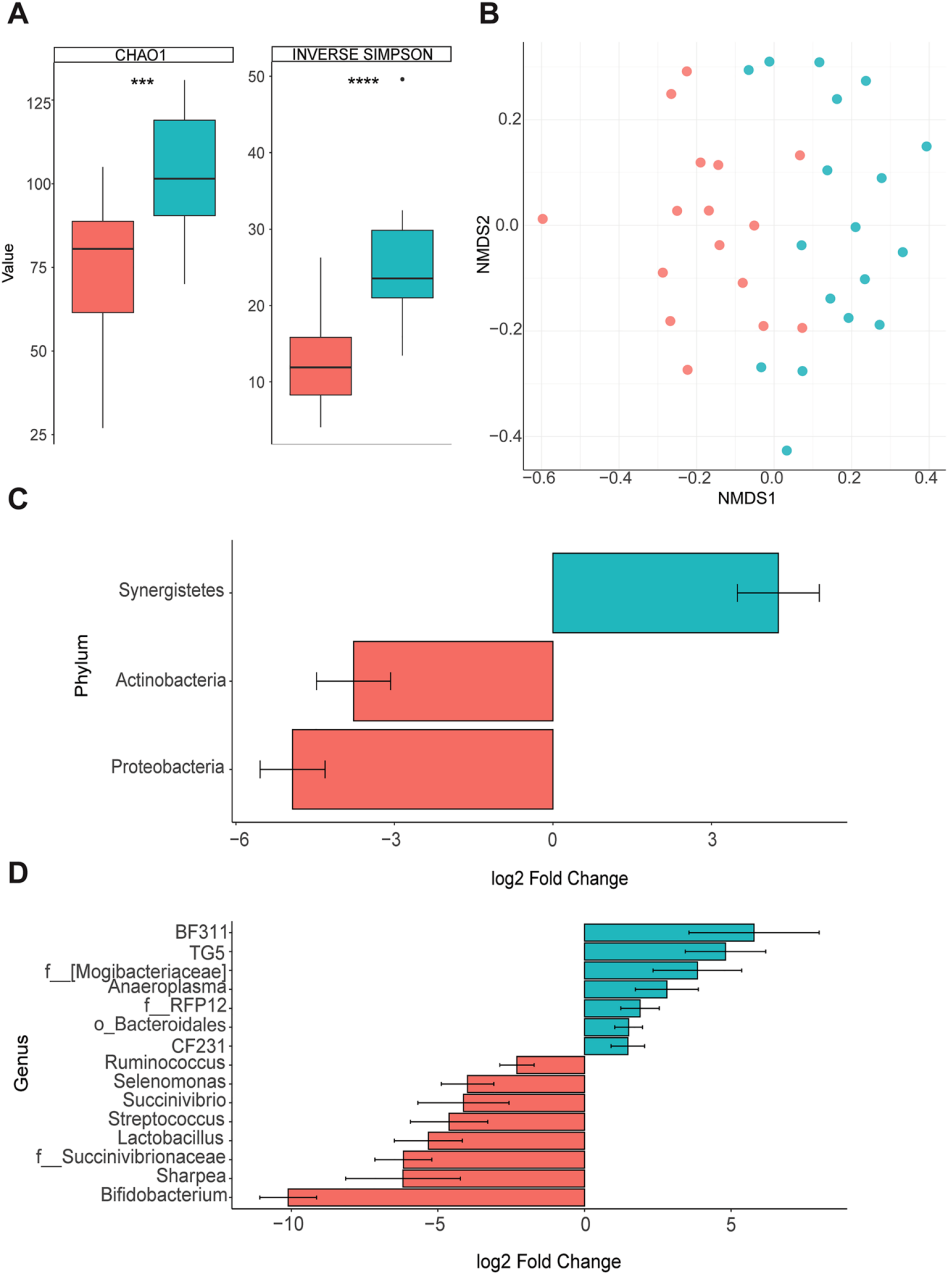
Measures of (**A**) Chao1 and Inverse Simpson alpha diversity and (**B**) Bray Curtis dissimilarities, and the Log2 fold change difference between significantly different (FDR *P*-value < 0.05) (**C**) phyla and (**D**) genera in the rumen fluid of control- (red) and carrot-fed (blue) lambs. Bray Curtis dissimilarities indicate separation (*P* = 0.01; PERMANOVA) of the rumen microbial community structure, where each dot represents one lamb. Significant differences are indicated by asterisks ***(*P* ≤ 0.001) and ****(*P* ≤ 0.0001). Positive fold change indicates greater abundance of the microbe in the rumen microbial community of carrot-fed lambs, whereas a negative fold change represents greater abundance of the microbe in control-fed lambs.

A total of 34 samples of rumen fluid and digesta were sequenced, resulting in 139,642 non-chimeric sequences after quality control and the identification of 2383 unique ASVs from all samples. Rarefaction analysis showed that sequencing depth was sufficient to capture most of the microbial diversity in the samples with rarefaction curves for all samples approaching an asymptote. Richness, based on the Chao1 index, and other alpha diversity metrics were greater (*P* < 0.01; Fig. 1A; Supplementary Fig. 1) for rumen bacteria from lambs fed the carrot diet. Based on the Bray–Curtis dissimilarity matrix, the carrot and control bacterial communities displayed distinct separation (*P* ≤ 0.01; PERMANOVA) from one another (Fig. 1B).

The three most dominant bacterial phyla comprised *Bacteroidetes* (63.0 ± 3.1%), *Firmicutes* (22.7 ± 2.3%) and *Synergistetes* (3.3 ± 1.3%) for carrot-fed lamb rumen microbial communities, and *Bacteroidetes* (51.9 ± 4.3%), *Firmicutes* (22.3 ± 3.7%) and *Proteobacteria* (14.9 ± 4.2%) for the control-fed lambs, respectively (Supplementary Table 1). However, *Synergistetes* was 4.26 ± 0.78 log2 fold higher (FDR-adjusted *P* ≤ 0.04) with the carrot diet, while *Actinobacteria* and *Proteobacteria* were greater with the control diet (FDR-adjusted *P* ≤ 0.01; Fig. 1C). The archaeal phylum, *Euryarchaeota* did not differ between treatments (FDR-adjusted *P* = 0.35; Supplementary Table 1).

*Prevotella* was the most dominant genus in the rumen of both carrot (42.0 ± 2.64%) and control (42.9 ± 3.49%) fed lambs and was unaffected by diet (FDR-adjusted *P* = 0.79; Supplementary Table 1). In the rumen bacterial community of carrot-fed lambs, an unclassified genus within the order *Bacteroidales* (15.9 ± 1.59%) and an unclassified genus of the *Veillonellaceae* family (9.11 ± 1.42) were second and third most dominant genera (FDR-adjusted *P* ≤ 0.01). However, in the rumen bacterial community of lambs fed the control diet, the second and third dominant genera included an unclassified genus of the *Succinivibrionaceae* family (11.1 ± 3.85%) and an unclassified genus of the order *Bacteroidales* (4.89 ± 0.95; adjusted *P* ≤ 0.01). Further, the relative abundance of unclassified *Succinivibrionaceae* was 72-fold lower (6.18 ± 0.97 log2 fold change; Fig. 1D) in the rumen bacterial community of lambs fed the carrot diet compared to the control lambs, while the relative abundance of the unclassified *Bacteroidales* was 2.8-fold higher (1.50 ± 0.47; Fig. 1D).

### Blood plasma, rumen and liver metabolome profile

**Figure 2:**
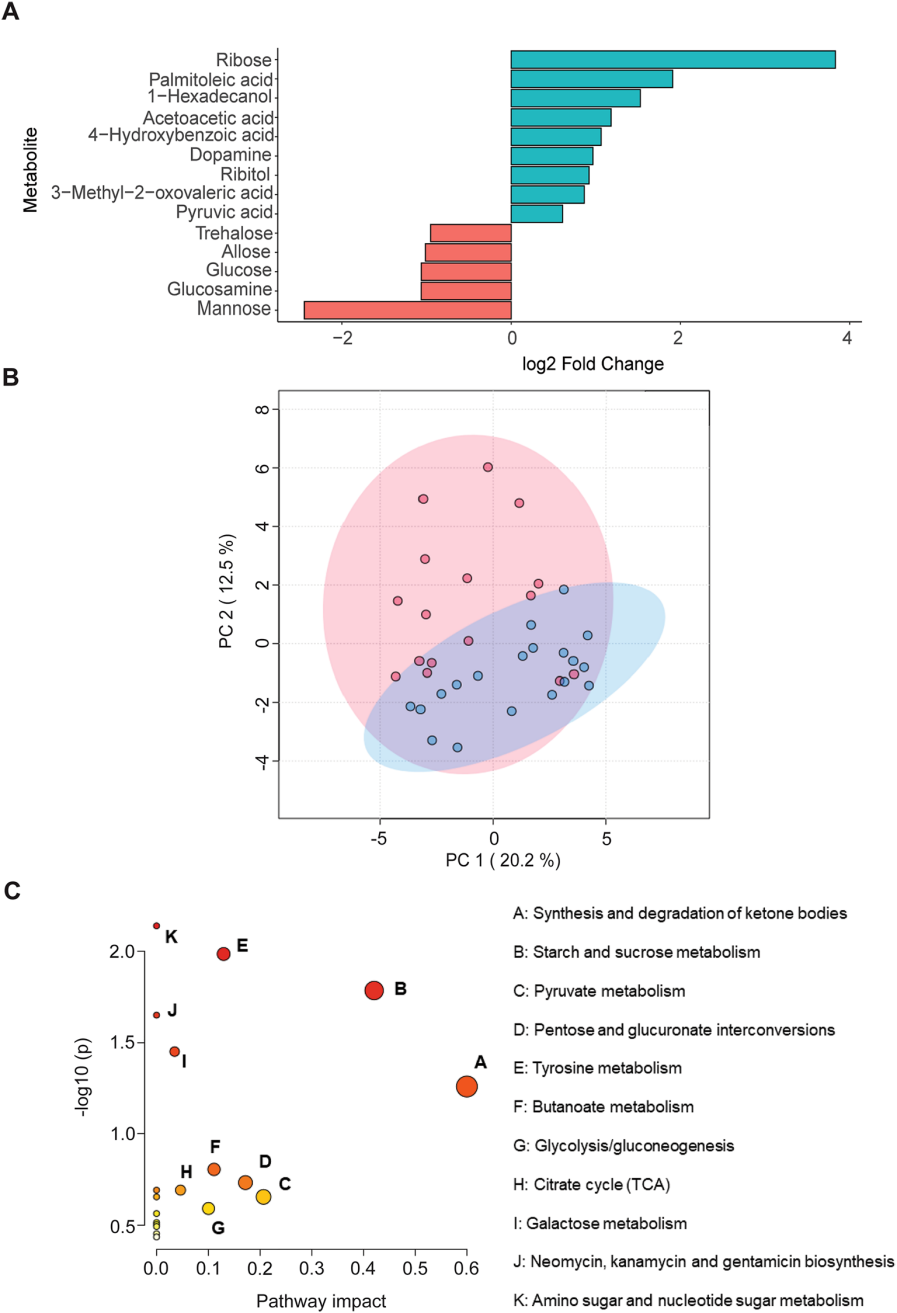
(**A**) Fold change differences between significantly different (FDR *P*-value < 0.05) rumen fluid metabolites. GC–MS identified fourteen diet-specific metabolites were detected and reflected by log fold change as determined by t-test (*P* < 0.05). Positive fold change indicates a greater proportion of the metabolite in the rumen fluid of carrot-fed (blue) lambs, while negative fold change represents a higher proportion in the rumen fluid of control-fed (red) lambs. Error bars were not included as data were normalised by median and log transformed. (**B**) Principal component analysis of metabolites that are significantly different by t-test (P < 0.05), where each data point represents one lamb. (**C**) Rumen fluid pathway enrichment analysis on MetaboAnalyst 5.0 associated with the *Bos taurus* KEGG pathway database for the influence of control or carrot diet on the rumen fluid metabolic profile. Pathways with an impact value ≥ 0.1 and *P*-value ≤ 0.05 are considered biologically relevant. Circle size indicates the magnitude of the pathway impact value, defined as the cumulative value of significant metabolites. Pathways of the greatest magnitude were labelled A–K, while colour is indicative of *P*-value, where red = *P* < 0.05 and ranges to white = *P* > 0.05 (Supplementary Table S2).

**Figure 3:**
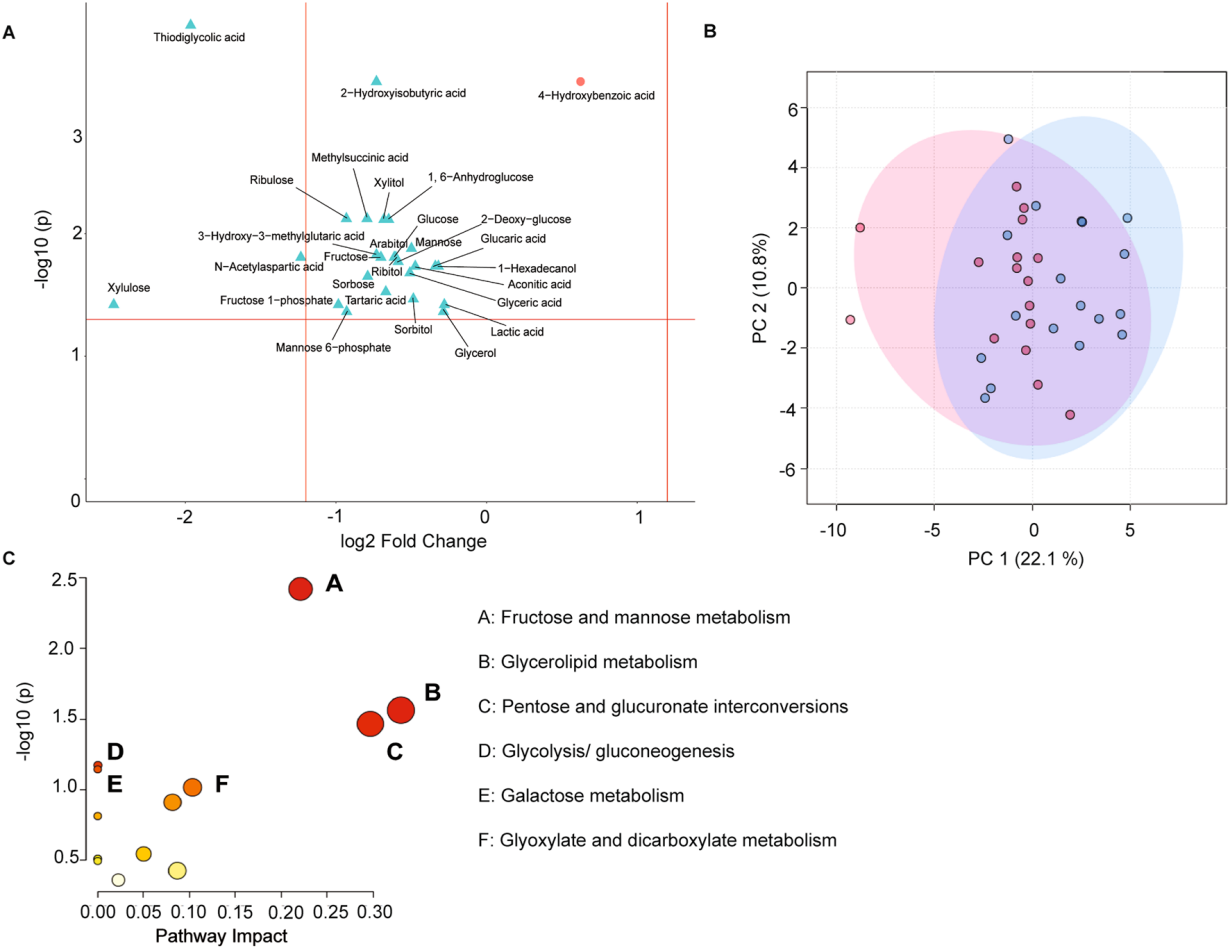
(**A**) Volcano plot of differentially expressed (FDR adjusted *P* ≥ 0.05) liver metabolites from lambs fed the carrot (red; positive log2 FC) and control (blue; negative log2 FC) diet. Twenty-seven liver metabolites were influenced by diet and reflected by log2 fold change as determined by t-test (*P* < 0.05). (**B**) Separation of liver metabolic profiles via principal component analysis for liver samples from control (red) and carrot-fed (blue) lambs. (**C**) Pathway analysis of liver metabolites enriched via MetaboAnalyst 5.0 associated with the *Bos taurus* KEGG pathways. Pathways (**A–C**) with an impact value ≥ 0.1 and *P*-value ≤ 0.05 are considered biologically relevant, and tendencies (0.05 ≥ *P* ≥ 0.10; (**D–F**)) were illustrated. Circle size indicates the magnitude of the pathway impact value, defined as the cumulative value of significant metabolites. Colour is indicative of *P*-value, where red = *P* < 0.05 and ranges to white = *P* > 0.05 (Supplementary Table S3).

Overall, 170 metabolites were detected in the blood plasma, 355 metabolites were detected in rumen fluid and 212 metabolites were detected in the liver. Univariate analyses of metabolite data and subsequent determination of statistically different metabolites by t-test resulted in the identification of 14 differentially expressed metabolites (*P* ≤ 0.05; Fig. 2A) in the rumen fluid, and 27 differentially expressed metabolites in the liver (*P* ≤ 0.05; Fig. 3A). Conversely, there was no influence of diet on the plasma metabolomics profile (FDR-adjusted *P* > 0.05; Supplementary Fig. 2).

Of the differentially expressed metabolites in rumen fluid, nine were more prevalent in the rumen fluid of lambs fed the carrot diet, compared to five with the control diet (Fig. 2A). There was clear clustering of the carrot samples from the control samples along the second principal component with the PCA analysis of the differentially expressed metabolites, explaining 12.5% of the variance between samples (Fig. 2B). An orthogonal partial least squares discriminant analysis (OPLS-DA), conducted on the total rumen fluid metabolites validated the diet-associated separation of metabolites by n = 100 permutations, which resulted in a *P* < 0.01 (Q^2^ = 0.76; R^2^ = 0.98; Supplementary Fig. 3A,B).

Pathway analyses using MetaboAnalyst 5.0 and the KEGG *Bos taurus* database (impact value ≥ 0.1; *P* ≤ 0.05; Fig. 2C) were conducted to identify the dietary influence on the involvement of these metabolites in metabolic pathways. Tyrosine metabolism (dopamine, acetoacetic acid and pyruvate; impact value = 0.13; *P* = 0.01; Fig. 2C; Supplementary Table 2) was more prevalent in rumen fluid from carrot-fed lambs. Moreover, the synthesis and degradation of ketone bodies (acetoacetic acid) tended (impact value = 0.6; *P* = 0.06) to be more prevalent in the rumen fluid of carrot-fed lambs. Contrastingly, starch and sucrose metabolism (trehalose and glucose) were the only metabolic pathway of biological relevance in the rumen fluid of control-fed lambs (impact value = 0.42; *P* ≤ 0.02).

Feeding the carrot diet resulted in the differential expression of twenty-seven liver metabolites between lambs fed the carrot and control diet. Interestingly, 4-hydroxybenzoic acid (FC = 0.62; FDR-adjusted *P* < 0.01; Fig. 3A) was the only liver metabolite influenced by the carrot diet, compared to 26 metabolites with the control diet (FDR-adjusted *P* ≤ 0.04). The differentially expressed liver metabolites were not strongly differentiated by the first or second principal component in a PCA (Fig. 3B).

In the enrichment analysis (Fig. 3C), 4-hydroxybenzoic acid was not included in any of the identified metabolic pathways. However, the fructose and mannose metabolism pathway (impact value = 0.22; *P* < 0.01; Supplementary Table 3) included sorbitol, mannose-6-phosphate and fructose-1-phosphate metabolites which had higher expression in the liver of lambs fed the control diet, as did glycerol and glyceric acid (glycerolipid metabolism pathway; impact value = 0.33; *P* = 0.03) and xylitol and xylulose (pentose and glucuronate interconversions pathway; impact value = 0.30; *P* = 0.03).

### Relationship between the microbial community, metabolic profile and functional pathways in rumen fluid

**Figure 4:**
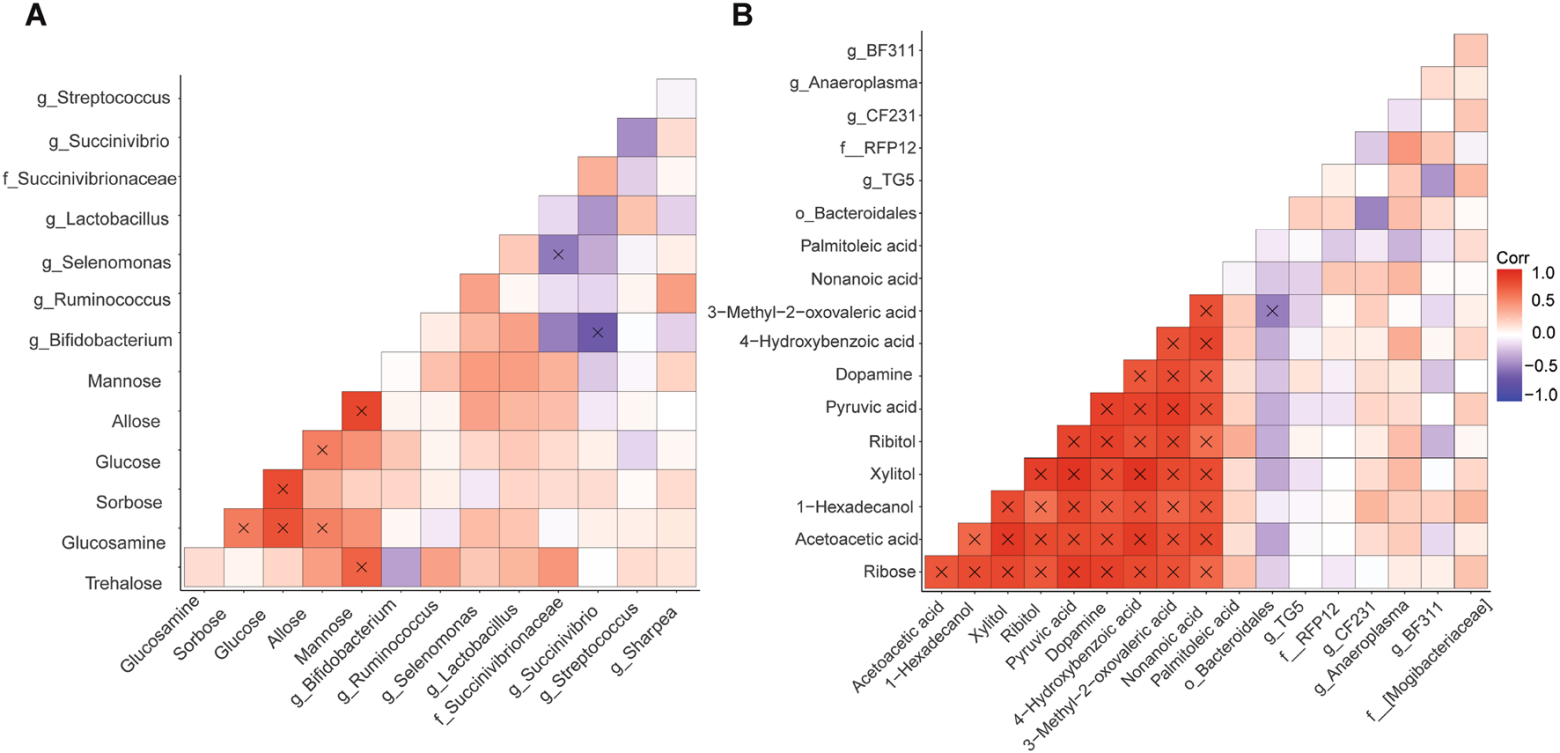
Spearman’s correlation of differentially expressed bacterial genera and metabolites detected in rumen fluid from lambs fed the (**A**) control or (**B**) carrot diet. Colour gradient of squares denotes a strong positive (red), strong negative (purple) or no (white) correlation, while “X” indicates a significant correlation (*P* ≥ 0.05).

Spearman’s correlations between all differentially expressed microbial genera and rumen fluid metabolites in the rumen fluid of carrot or control-fed lambs determined 9 significant correlations in rumen fluid collected from control-fed lambs (*P* ≤ 0.05; Fig. 4A). Conversely, 45 significant correlations were determined between metabolites, and 1 between the microbial community and rumen fluid metabolites from carrot-fed lambs (*P* ≤ 0.05; Fig. 4B). An unclassified taxon within the family *Succinivibrionaceae* was negatively correlated (R^2^ =  − 0.51; *P* = 0.04) with *Selenomonas* in the rumen fluid of control-fed lambs, while *Bifidobacterium* was negatively correlated with *Succinivibrio* (R^2^ =  − 0.66; *P* < 0.01). However, all other significant correlations occurred between rumen metabolites in control-fed lambs (R^2^ ≥ 0.58; *P* ≤ 0.04).

Across genera that were differentially expressed with the carrot diet, an unclassified genus within the order *Bacteroidales* was negatively correlated with 3-methyl-2-oxovaleric acid (R^2^ ≥  − 0.49; *P* = 0.04; Fig. 4B), while no other bacterial genera were significantly correlated with one another or metabolites analysed (*P* ≥ 0.05). However, with the exception of palmitoleic acid, all rumen metabolites were positively correlated with one another (R^2^ ≥ 0.63; *P* ≤ 0.04).

**Figure 5:**
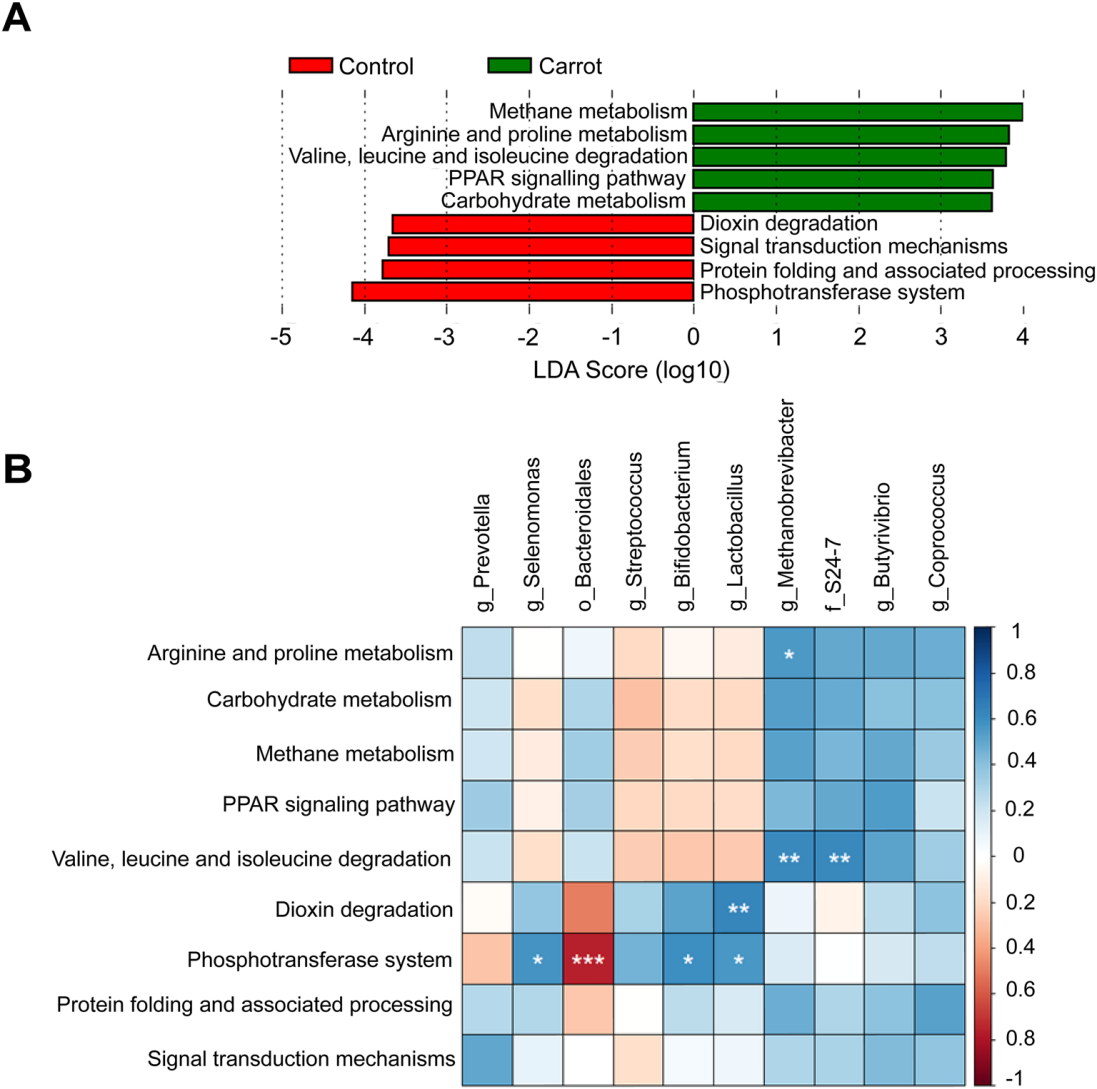
(**A**) Linear discriminant analysis of the control and carrot diet. LDA scores ≥ 2 indicate the metabolic pathways that may best explain differences observed between diet treatments and (**B**) Correlation matrix of rumen bacterial and archaeal genera and metabolic pathways identified through CowPI and linear discriminant analysis that were positively (blue) or negatively (red) correlated (− 1 ≤ R^2^ ≤ 1). Asterisks (*) indicate level of significance, where ***P ≤ 0.001; **P ≤ 0.01 and *P ≤ 0.05.

Among significant pathways (LDA log score ≥ 3.62; Fig. 5A), methane, arginine and proline metabolism, valine, leucine and isoleucine, PPAR signalling pathway and carbohydrate metabolism were upregulated in the carrot diet determined through microbial function prediction. Conversely, dioxin degradation, signal transduction mechanisms, protein folding and associated processing and the phosphotransferase system were functional pathways upregulated in the control diet compared to the carrot diet (LDA score log score ≥ 3.70). The phosphotransferase system was negatively correlated (R^2^ =  − 0.75; *P* < 0.001; Fig. 5B) with ASVs within *Bacteroidales*, while *Selenomonas*, *Bifidobacterium* and *Lactobacillus* were positively correlated (R^2^ ≥ 0.56; *P* ≤ 0.04).

Moreover, *Methanobrevibacter* was more abundant with the carrot diet. Branch chained amino acid (valine, leucine and isoleucine) degradation (R^2^ = 0.61; *P* ≤ 0.01), as well as arginine and proline metabolism (R^2^ = 0.56; *P* ≤ 0.001) pathways were positively correlated with *Methanobrevibacter*. Further, unclassified family S24-7 was significantly correlated with valine, leucine and isoleucine degradation (*P* ≤ 0.01), corresponding with a negative correlation between 3-methyl-2-oxovaleric acid and *Bacteroidales* (Fig. 5B).

## Discussion

To our knowledge, this is the first study to utilise comprehensive, targeted metabolomics approach via GC–MS to evaluate the contribution of the rumen microbiota and metabolic profiles to improvements in rumen function and provide a possible explanation to the superior lamb performance^12^ observed by feeding an unsalable carrot TMR. However, while this study profiled the rumen microbial, plasma and liver metabolites, ruminal tissue metabolites were not profiled. Therefore, functional prediction analyses were conducted utilising the CowPI platform for rumen microbes to predict rumen-associated metabolic pathways^40^, providing information which may explain the noted superior lamb performance.

The rumen microbial environment comprises a core microbiome, and a consortium of microbes that are readily modified by diet^26^, seen through the dissimilarity of carrot and control-fed microbial communities in this study. Prior reports have also indicated that diet influences the rumen metabolome, as a grain-based TMR and pasture feeding have shifted their microbial and metabolic profiles, respectively^41,42^. The replacement of barley grain with unsalable carrots at 45% DM resulted in a greater Bray–Curtis distance and greater α-diversity among the rumen archaeal and bacterial consortia, while consequently modifying the rumen metabolic profile.

The predominant phyla detected irrespective of diet in this study were *Bacteroidetes* and *Firmicutes*. In addition, the relative abundance of an unclassified genus within the order *Bacteroidales* was increased by 1.50 ± 0.47 log2 fold in the rumen fluid of carrot-fed lambs, compared to the control. Knowledge on the taxonomy, function and metabolism of this genus remains uncertain. Although, recent reports have indicated that *Bacteroides thetaiotaomicron*, a member of *Bacteroidales*, utilises ribose (d-ribose)^43^ as a source of carbon and energy for growth. Ribose, found within the lipo- or polysaccharide components of bacterial cell walls^44^ was greater in the rumen fluid of carrot fed lambs, was negatively correlated with *Bacteroidales*, and has similarly been detected in hay-concentrate diets^45^. Therefore, it is possible that the unclassified *Bacteroidales* utilised ribose as a substrate for cellular maintenance given its greater relative abundance in the microbial community of carrot fed lambs. *Bacteroidales* may have contributed to feed efficiency, as it was up to 39% higher in the microbial communities of bulls with greater feed efficiency^46,47^, while similarly increasing by 25% in lambs fed the carrot diet^12^.

Through the CowPI workflow, a negative correlation (R^2^ =  − 0.75; *P* < 0.001) was found between the order *Bacteroidales* and the phosphotransferase system. This is consistent with findings by Ref.^48^, as some species within the order *Bacteroidales* do not possess a phosphotransferase system for mannose utilisation. Complementing this, high-level functional interpretation of biological samples inferred a negatively correlated (R^2^ ≥  − 0.49; *P* = 0.04) relationship between *Bacteroidales* and 3-methyl-2-oxovaleric acid, a metabolite of isoleucine produced during branch chain amino acid degradation^49^ that wasdetected in the rumen fluid of carrot-fed lambs.

In addition, unclassified *Bacteroidales* were negatively correlated with 4-hydroxybenzoic acid, which may indicate a possible route of utilisation, due to the presence of 4-hydroxybenzoate carboxylase in some facultative anaerobic bacteria^50^. Consequently, this was also the only metabolite in the liver metabolome of carrot-fed lambs which differed to control-fed lambs. It is possible that greater 4-hydroxybenzoic acid in the liver metabolome of carrot-fed lambs was the result of passive exchange of metabolites between the rumen epithelia and portal blood streams, as previously observed with metabolites such as propionate^51^. Consequently, 4-hydroxybenzoic acid may have been rapidly hydrolysed into benzoic acid and metabolised in the lamb liver, similar to the metabolism of parabens in the human liver^52^. Further, carrot cell walls are naturally rich in 4-hydroxybenzoic acid^53,54^ which accumulates in its esterified form through direct contact of carrots with soil minerals prior to harvest^55,56^. In this study, carrots were chopped prior to feeding, thereby damaging the carrot cell wall such that accumulation of 4-hydroxybenzoic acid may have occurred in the rumen of carrot-fed lambs. This may provide a possible explanation as to the differential expression of 4-hydroxybenzoic acid in the rumen fluid, and subsequent accumulation in the liver.

The presence of 4-hydroxybenzoic acid in the rumen environment, has previously reduced cellulolytic activity of fibrolytic *Fibrobacter succinogenes* by up to 38%, while promoting growth of *Ruminococcus* species^57–59^. Further, Jung et al.^60^ noted that 4-hydroxybenzoic acid content in lucerne hay increased with lignification by up to 46% between bud and full bloom. This corresponded with a decrease in rumen digestibility, thus accumulation of 4-hydroxybenzoic acid in the rumen of cannulated sheep. Although, a higher abundance of *Ruminococcus* in the rumen fluid of control-fed lambs in this study was possibly associated with the greater proportion of lucerne hay, thus DM and NDF content in the control compared to the carrot diet^12^. Instead, fibre within the carrot diet could have undergone degradation via *Fibrobacter* or by anaerobic fungi and protozoa^61,62^, which were not characterised in this study. However, our findings demonstrated that the substitution of barley grain for carrot may have coincided with a change in the source of rapidly degradable substrates available to rumen microbes.

In this study, a 20.5% increase in the molar proportion of propionate in the control diet stem from the use of steam-flaked barley grain—up to 78% of which is rumen degradable^63^. This increases available succinate for conversion into propionate by rumen bacterial genera including *Succinivibrio* and *Roseburia*^64,65^, which were up to 4.13 log fold more abundant in the rumen fluid of control-fed lambs. Starch degradation pathways are utilised by amylolytic rumen bacteria^66^ such as *Succinivibrio*, which had a strong positive correlation with propionate when feeding a starch-rich, grain-based diet^67^. Although, Hatew et al.^68^ ascertained that the type of grain posed a greater influence on the molar proportion of propionate than starch content. Barley grain is a conventional concentrate rich in starch which rapidly degrades to glucose in the rumen^69^, and was most prevalent in the control diet.

Amylolytic taxa within the *Succinivibrionaceae* family (11.1 ± 3.85% RA), *Lactobacillus* (2.2 ± 0.90%) and *Streptococcus* (2.3 ± 1.57%) were differentially expressed in the rumen fluid of control-fed lambs, aligning with prior reports detailing the increased abundance of these genera when a grain diet was fed to cattle^70,71^. Further, functional analysis determined that the carbohydrate phosphotransferase system, associated with the transport of carbohydrates such as mannose was more prevalent in the rumen fluid of control-fed lambs and is a method of carbohydrate uptake utilised by taxa including *Selenomonas*, *Bifidobacterium* and *Lactobacillus*^48^. Supporting these findings, Spearman correlation of *Bifidobacteria* and unclassified *Succinivibrionaceae* with trehalose suggests possible utilisation and production, respectively, as both genera are involved in starch and sucrose metabolism in the rumen of sheep and cattle^42,71^.

Moreover, the unclassified *Succinivibrionaceae* was also positively correlated with *Selenomonas* in rumen fluid from control-fed lambs, which typically decarboxylates succinate, produced by *Succinivibrionaceae* into propionate by *Selenomonas*^71^. *Selenomonas* taxa present in the rumen are associated with the production of acetate, propionate and lactate^72^, while lactate is used as a growth substrate in others^73,74^. Several results in this study, including the proportion of molar propionate or the reduction in rumen pH for control-fed lambs could have been the result of differential expression of *Selenomonas*. As the rumen pH declines, the proportion of lactic-acid producing bacteria increases, while the proportion of lactic-acid utilising bacteria simultaneously decreases^75^. However, taxa within the *Sharpea* genus are minor lactic acid-producing bacteria^76^ that were differentially expressed in the control diet. These genera possibly contributed to the lower observed in vivo rumen pH, as they have previously been detected in cattle under acidotic challenge when fed high-grain diets^77^.

Generally, a grain-based diet high in starch will inhibit methanogen activity, thus reducing CH_4_ emissions by mode of an increase in molar propionate and a decrease in rumen pH. Interestingly, fermentation via in vitro batch culture of the carrot diet resulted in a reduction of CH_4_ concentration by 13.8% on an mg/g DM basis, despite there being a numerically greater (*P* = 0.11) proportion of *Methanobrevibacter* in the rumen fluid of carrot-fed lamb. A reduction in CH_4_ production was not associated with an effect of diet on gas production nor the percentage of CH_4_. However, Tapio et al.^78^ proposed that the composition, rather than the abundance of methanogenic archaea influences the intensity of CH_4_ emissions, with *Methanobrevibacter ruminantium* and *Methanobrevibacter gottschalkii* having been noted as more abundant in ruminants with low CH_4_ emissions and high CH_4_ emissions, respectively^79^. Prior reports have also indicated that *Methanosphaera* was negatively correlated (R^2^ =  − 0.47; *P* < 0.02) with CH_4_ production, and tended to be 4.48 log_2_ fold more abundant in the rumen fluid of carrot-fed lambs^80^. Hydrogenotrophic species within *Methanobrevibacter* may metabolise amino and branched chain amino acids into organic nitrogen in order to proliferate in the rumen environment^81,82^. This could also be due to the mutualistic relationship of *Methanobrevibacter* with rumen bacteria^83^, promoting the oxidation of substrates for use by proteolytic bacteria such as Prevotella^84^ by utilising free H_2_. However, with the carrot diet, predicted in vivo CH_4_ yield was numerically lower than the control by 13.1% indicating that the carrot diet may affect fermentation by rumen microbes and resultant CH_4_ yield.

Dietary intervention^85,86^ is a CH_4_ mitigation strategy that involves the reduction of ruminal CH_4_ through dietary unsaturated fatty acids, linoleic (n6-18:2; LA), α-linolenic (n6-18:3; ALA) and oleic acids (c9-18:1) as terminal acceptors of H_2_^87–89^. Unclassified ASVs within the order *Bacteroidales* were linked via MiCA terminal restriction fragments to conjugated linoleic acid as well as saturated fatty acids t11-18:1 and 18:0, indicating a possible role in ruminal biohydrogenation^90^. Fatty acid biohydrogenation in the rumen is primarily driven by members of *Butyrivibrio*^91,92^. However, biohydrogenating taxa isomerise cis-12 bonds and hydrogenated double bonds in conjugated LA to produce stearic acid (18:0)^93^. A negative correlation between the unclassified *Bacteroidales* taxa and 18:0, Dewanckele et al.^91^ suggests a similar capacity for biohydrogenation, as the carrot diet contained 46.4% and 10.6% more total mono- and poly-unsaturated fatty acid substrate compared to the control diet, respectively^12^. This could possibly explain the decrease in in vitro CH_4_ production using the carrot diet as fermentation substrate, but future studies should ascertain the specific role of the unclassified members within *Bacteroidales* to better understand CH_4_ mitigation via microbial biohydrogenation.

The phylum *Synergistetes* include anaerobic bacteria that are unable to metabolise sugars, thus obtain energy via the fermentation of aromatic amino acids^94,95^. The genus *TG5,* of the *Synergistetes* phylum was more abundant in the rumen fluid of carrot-fed lambs, which coincided with the differential expression of acetoacetic acid, a product of ketogenic or glucogenic amino acid catabolism^96^. Connected to tyrosine metabolism via pathway analysis, acetoacetic acid was detected in the rumen fluid of carrot-fed lambs and is a product of tyrosine degradation^97^, resulting in a ketone body that completes the tyrosine degradation pathway via its influence on fumarylacetoacetate hydrolase^98^. Similarly, 3-methyl-2-oxovaleric acid, a product of isoleucine metabolism^99^ was also greater in the rumen fluid of carrot-fed lambs and was negatively correlated with the unclassified *Bacteroidales*. This suggests a possible route of utilisation for *Bacteroidales*, as the taxa does not secrete 3-methyl-2-oxovaleric acid and has previously been associated with branched chain amino acid metabolism^100,101^.

## Conclusions

Substituting barley grain for unsalable carrots at 45% DM increased rumen pH by 8.5%, improved in vitro diet digestibility by 8.8% and reduced in vitro CH_4_ by 13.8% on an mg/g DM basis. The rumen fluid of carrot-fed lambs diverged from control-fed lambs as indicated by a greater Bray–Curtis distance, confirming our hypothesis. Further, a diet-associated shift in the microbiome-metabolome relationship could be seen with the detection of 14 treatment-specific rumen fluid metabolites, and the sole liver metabolite, 4-hydroxybenzoic acid being greater in carrot-fed lambs. Moreover, our results provide evidence toward the possible roles of unclassified taxa within *Bacteroidales* in improving feed efficiency and fatty acid biohydrogenation, and a potential role of *TG5* amino acid metabolism. Such relationships between metabolites that are utilised or produced by the rumen microbiome may explain previously observed improvements in feed efficiency and animal performance. Therefore, replacing barley grain with unsalable carrots at 45% DM is an effective means of eliciting animal performance improvements beyond that of conventional feedlot rations by shifting the rumen microbial and metabolic profiles.

## Supplementary Information

Supplementary Information.

## Data Availability

All 16S rRNA gene sequences were submitted to the NCBI Sequence Read Archive under BioProject accession number PRJNA772293.
