# Supplementary material for: Feeding an unsalable carrot total-mixed ration altered bacterial amino acid degradation in the rumen of lambs
Source: Sci Rep. 2023 Apr 28;13:6942. doi: 10.1038/s41598-023-34181-0 (PMC10147942; doi:10.1038/s41598-023-34181-0)
Supplement: Supplementary file 1 — Supplementary Information. [file 41598_2023_34181_MOESM1_ESM.pdf]

Supplementary Material

Feeding an unsalable carrot total-mixed ration altered bacterial amino acid degradation in the rumen of lambs

Daniel L. Forwood, David J. Innes, Mariano C. Parra, Terra Stark, David P. de Souza, Alex V. Chaves, Sarah J. Meale

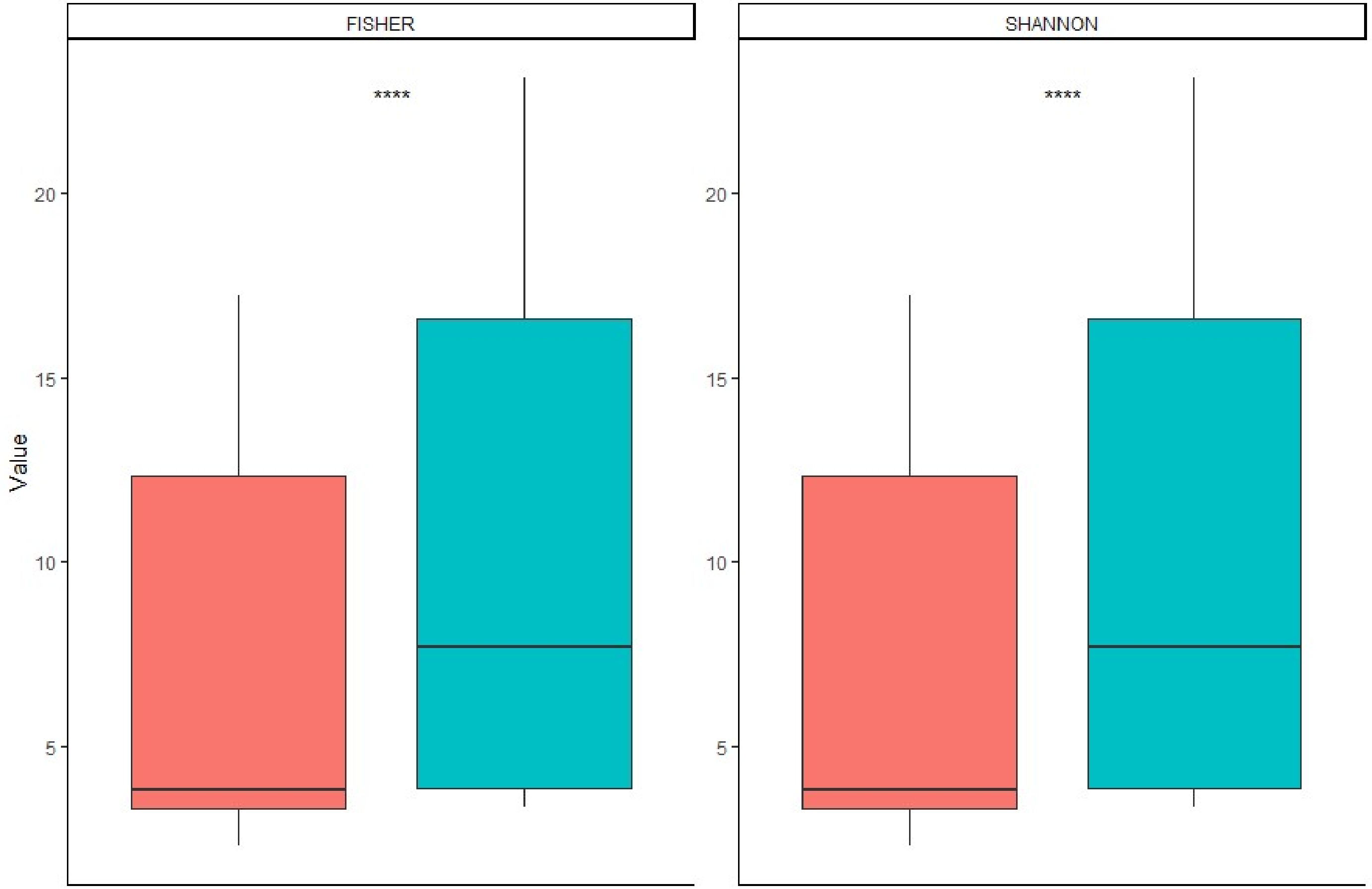

Supplementary Figure 1. Measures of Fisher and Shannon alpha-diversities in the rumen fluid of lambs fed the control (red) and carrot (blue) diet. Asterisks indicate significant differences between diets (**; P ≤ 0.0001).**

Supplementary Table 1. Percent relative abundance (PRA) and log2 fold changes of archaeal and bacterial taxa detected in the rumen fluid from carrot and control-fed lambs. A positive log2 fold change suggests a higher abundance in the carrot diet, while a negative log2 Fold change suggests a greater abundance in the control diet. Significance was declared if adjusted P-value ≤ 0.05.

	Control PRA	Control SEM	Carrot PRA	Carrot SEM	Base mean	log2 FC	log2 FC SEM	Adjusted P-value
**Archaea**								
**Euryarchaeota**	**1.26**	**0.32**	**3.09**	**0.87**	126.9	0.71	0.47	0.35
Methanobrevibacter	1.26	0.32	3.18	0.96	125.9	1.04	0.51	0.11
Methanosphaera	n.d.	n.d.	0.08	0.03	2.20	4.48	2.08	0.09
Methanomassiliicoccaceae - F	0.004	0.004	0.03	0.01	0.97	2.22	2.63	N/A
Methanomassiliicoccaceae vadinCA11	0.005	0.005	0.18	0.07	4.72	4.23	1.78	0.06
**Bacteria**								
**Actinobacteria**	**5.37**	**1.79**	**0.38**	**0.09**	198.5	-3.77	0.70	<0.01
Bifidobacterium	3.84	1.56	n.d.	n.d.	97.8	-10.11	0.97	<0.01
Coriobacteriaceae - F	1.52	0.43	n.d.	n.d.	63.0	-1.71	0.79	0.09
Adlercreutzia	0.01	0.01	0.01	0.01	0.29	-0.55	2.97	N/A
Atopobium	0.05	0.03	0.02	0.01	1.83	-1.60	2.56	0.69
**Bacteroidetes**	**51.9**	**4.31**	**63.0**	**3.11**	4658.8	-0.15	0.33	0.76
Bacteroidales - O	4.89	0.95	15.86	1.59	803.7	1.50	0.47	0.01
Paraprevotellaceae - F	0.48	0.12	1.18	0.20	61.2	0.88	0.61	0.31
[Prevotella]	0.28	0.12	0.09	0.03	12.0	-1.41	1.47	0.53
Paraprevotellaceae CF231	0.74	0.20	2.18	0.29	102.7	1.48	0.57	0.04
Paraprevotellaceae YRC22	0.70	0.17	0.48	0.11	43.9	-0.86	0.63	0.35
Bacteroidaceae BF311	n.d.	n.d.	0.21	0.09	5.44	5.78	2.22	0.04
Paludibacter	0.10	0.06	0.46	0.16	17.8	2.26	1.49	0.28
Prevotellaceae - F	0.27	0.12	1.27	0.32	52.6	2.01	1.00	0.11
Prevotella	42.9	3.49	42.0	2.64	3265.9	-0.15	0.34	0.79

Bacteroidales RF16 - F	1.05	0.24	1.88	0.46	103.1	0.72	0.71	0.51
Bacteroidales S24-7 - F	0.79	0.31	1.52	0.24	78.8	1.22	0.58	0.1
**<u>Chloroflexi</u>**	**0.05**	**0.03**	**0.10**	**0.05**	4.85	1.09	1.68	0.75
Anaerolinaceae SHD-231	0.05	0.03	0.10	0.05	4.58	1.23	1.69	0.64
**<u>Cyanobacteria</u>**	**0.09**	**0.03**	**0.26**	**0.10**	8.66	0.46	1.07	0.76
YS2 - O	0.09	0.03	0.28	0.11	11.3	1.22	1.13	0.48
**<u>Elusimicrobia</u>**	**0.01**	**0.01**	**0.15**	**0.07**	3.44	3.45	2.16	0.35
Elusimicrobiales - O	n.d.	n.d.	0.06	0.06	0.00	0.00	0.00	N/A
Elusimicrobiaceae - F	0.01	0.01	0.05	0.04	0.88	1.24	2.94	N/A
Endomicrobia - C	n.d.	n.d.	0.06	0.04	0.73	2.89	2.95	N/A
**<u>Fibrobacteres</u>**	**2.06**	**0.77**	**2.52**	**0.57**	171.2	-0.14	0.61	0.87
Fibrobacter	2.08	0.77	2.72	0.60	191.2	-0.21	0.67	0.82
**<u>Firmicutes</u>**	**22.3**	**3.67**	**22.7**	**2.34**	1585.7	-0.15	0.28	0.76
Enterococcus	0.03	0.03	n.d.	n.d.	0	0.00	0.00	N/A
Lactobacillus	2.22	0.90	0.04	0.04	56.6	-5.33	1.16	<0.01
Streptococcus	2.29	1.57	0.04	0.02	33.8	-4.62	1.32	<0.01
Clostridiales - O	1.71	0.34	3.07	0.63	161.8	0.43	0.52	0.61
Mogibacteriaceae - F	0.01	0.01	0.10	0.03	2.97	3.85	1.51	0.04
Mogibacterium	0.15	0.05	0.05	0.03	5.32	-1.60	1.43	0.47
Clostridiaceae 02d06	0.24	0.12	0.03	0.03	6.68	-2.19	2.09	0.5
Clostridiaceae_Clostridium	0.22	0.09	0.38	0.08	20.8	0.57	0.98	0.71
Psuedoramibacter_Eubacterium	0.04	0.02	n.d.	n.d.	1.39	-0.94	2.93	N/A
Lachnospiraceae - F	0.22	0.07	0.54	0.11	23.0	1.21	0.78	0.27
Anaerostipes	0.04	0.04	0.02	0.02	0.00	0.00	0.00	N/A
Blautia	n.d.	n.d.	0.04	0.03	0.28	1.65	2.97	N/A

Butyrivibrio	0.61	0.21	0.59	0.13	38.2	-0.07	0.88	0.96
Lachnospiraceae_Clostridium	0.08	0.04	n.d.	n.d.	1.44	-4.03	2.71	N/A
Coprococcus	0.38	0.14	0.26	0.09	21.4	-0.88	1.22	0.64
Moryella	n.d.	n.d.	0.02	0.02	0.16	1.03	2.97	N/A
Oribacterium	0.03	0.02	0.01	0.01	0.72	-3.03	2.95	N/A
Pseudobutyrivibrio	0.14	0.05	0.14	0.04	9.10	0.36	1.35	0.83
Roseburia	0.24	0.16	n.d.	n.d.	0.06	-0.73	2.97	N/A
Shuttleworthia	0.06	0.04	0.02	0.02	1.57	0.09	2.92	0.98
Peptostreptococcaceae_Clostridium	0.005	0.005	0.04	0.04	0.19	-1.45	2.97	N/A
Peptostreptococcus	0.01	0.01	n.d.	n.d.	0.07	-0.77	2.97	N/A
Ruminococcaceae - F	0.21	0.04	0.26	0.11	14.0	0.26	0.80	0.82
Ruminococcaceae_Clostridium	n.d.	n.d.	0.08	0.06	0.63	2.67	2.95	N/A
Oscillospira	0.28	0.13	0.40	0.12	22.5	0.45	1.19	0.8
Ruminococcus	2.93	0.59	0.58	0.07	112.6	-2.30	0.59	<0.01
Veillonellaceae - F	3.40	1.30	9.11	1.42	438.8	1.65	0.73	0.08
Anaerosinus	n.d.	n.d.	0.07	0.07	0.00	0.00	0.00	N/A
Anaerovibrio	0.04	0.02	0.10	0.06	1.73	-1.92	2.93	0.68
Megasphaera	0.05	0.04	n.d.	n.d.	1.56	-4.11	2.94	0.33
Mitsuokella	0.10	0.07	n.d.	n.d.	0.00	0.00	0.00	N/A
Schwartzia	0.08	0.06	0.01	0.01	1.82	-2.56	2.83	0.56
Selenomonas	3.30	0.79	0.17	0.07	104.1	-3.99	0.89	<0.01
Succiniclasticum	1.39	0.33	1.18	0.14	89.3	-0.31	0.42	0.64
Veillonella	0.92	0.92	n.d.	n.d.	0.00	0.00	0.00	N/A
Bulledia	0.03	0.02	0.04	0.03	0.93	-1.54	2.94	N/A
Erysipelotrichaceae RFN20	0.05	0.03	0.04	0.01	2.67	-0.59	1.43	0.79
Sharpea	0.23	0.11	n.d.	n.d.	6.48	-6.19	1.96	<0.01
**Planctomycetes**	**0.05**	**0.02**	**0.12**	**0.04**	5.11	0.95	1.29	0.75
Pirellulaceae - F	0.05	0.02	0.14	0.05	5.29	1.54	1.27	0.41

**Proteobacteria**	**14.9**	**4.24**	**0.80**	**0.12**	743.7	-4.93	0.62	<0.01
Alphaproteobacteria - C	n.d.	n.d.	0.01	0.01	0.33	1.80	2.96	N/A
Alphaproteobacteria RF32 - O	0.04	0.02	0.09	0.03	4.72	0.67	1.60	0.79
Rickettsiales - O	0.02	0.01	0.04	0.03	1.70	0.66	2.35	0.83
Sutterella	0.002	0.002	0.02	0.01	0.66	1.96	2.75	N/A
Kingella	n.d.	n.d.	0.01	0.01	0.49	2.31	2.96	N/A
Desulfovibrio	0.01	0.01	0.01	0.01	0.46	0.63	2.96	N/A
Deltaproteobacteria GMD14H09 - O	n.d.	n.d.	0.01	0.01	0.15	1.13	2.97	N/A
Myxococcales 0319-6G20 - F	0.01	0.01	n.d.	n.d.	0.14	-1.21	2.97	N/A
Campylobacter	n.d.	n.d.	0.01	0.01	0.17	1.18	2.97	N/A
Succinivibrionaceae - F	11.1	3.85	0.33	0.12	633.8	-6.18	0.97	<0.01
Ruminobacter	1.63	1.02	0.08	0.03	27.8	-3.39	1.71	0.11
Succinivibrio	2.30	1.08	0.15	0.08	103.8	-4.13	1.56	0.04
Escherichia	0.004	0.004	0.05	0.03	1.71	3.16	2.49	0.39
**Spirochaetes**	**1.13**	**0.57**	**1.10**	**0.27**	53.5	0.48	0.69	0.75
MVP-15 PL-11B10 - O	n.d.	n.d.	0.02	0.02	0.00	0.00	0.00	N/A
Spirochaetaceae - F	n.d.	n.d.	0.07	0.03	2.05	4.38	2.08	0.1
Treponema	1.14	0.58	1.10	0.29	54.1	0.59	0.74	0.62
**SR1**	**0.06**	**0.04**	**0.01**	**0.01**	1.42	-2.31	2.40	0.67
SR1 - P	0.07	0.04	0.01	0.01	1.56	-1.63	2.34	N/A
**Synergistetes**	**0.19**	**0.10**	**3.96**	**1.35**	131.6	4.26	0.78	<0.01
Pyramidobacter	0.06	0.02	0.10	0.03	5.44	0.65	1.23	0.74
Dethiosulfovibrionaceae TG5	0.13	0.10	4.12	1.53	150.9	4.81	1.37	<0.01
**Tenericutes**	**0.08**	**0.03**	**0.24**	**0.09**	8.07	1.00	0.81	0.5
Anaeroplasma	0.02	0.01	0.24	0.09	7.11	2.81	1.07	0.04
Mollicutes RF39 - O	0.06	0.03	0.01	0.01	1.45	-1.75	2.08	N/A

**<u>Verrucomicrobia</u>**	**0.32**	**0.07**	**1.50**	**0.41**	48.4	1.41	0.58	0.06
Opitutae HA64 - O	0.003	0.003	0.02	0.01	0.47	1.12	2.96	N/A
WCHB1-41 RFP12 - F	0.30	0.07	1.55	0.42	57.8	1.89	0.66	0.02
WCHB1-41 WCHB1-25 - F	0.02	0.01	0.02	0.01	1.57	0.46	2.14	0.86
**<u>WPS-2</u>**	**0.04**	**0.03**	**0.01**	**0.01**	0.23	-0.07	2.97	0.98
WPS-2 - P	0.04	0.03	n.d.	n.d.	0.29	0.54	2.97	N/A

PRA, percent relative abundance; SEM, standard error of the mean; C, Class; F, Family; O, Order; FC, fold change; n.d., not detected

Supplementary Table 2. Pathway analysis of rumen fluid metabolites detected in lambs fed a carrot or control diet.

Pathway	Total	Hits	Raw p	-log10(p)	Holm adjust	FDR P-Value	Impact
Synthesis and degradation of ketone bodies	5	1	0.06	1.26	1.00	0.77	0.60
Starch and sucrose metabolism	18	2	0.02	1.79	1.00	0.46	0.42
Pyruvate metabolism	22	1	0.22	0.65	1.00	1.00	0.21
Pentose and glucuronate interconversions	18	1	0.19	0.73	1.00	1.00	0.17
Tyrosine metabolism	42	3	0.01	1.99	0.86	0.43	0.13
Butanoate metabolism	15	1	0.16	0.81	1.00	1.00	0.11
Glycolysis / Gluconeogenesis	26	1	0.26	0.59	1.00	1.00	0.10
Citrate cycle (TCA cycle)	20	1	0.20	0.69	1.00	1.00	0.05
Galactose metabolism	27	2	0.04	1.45	1.00	0.60	0.03
Amino sugar and nucleotide sugar metabolism	37	3	0.01	2.14	0.61	0.43	0.00
Neomycin, kanamycin and gentamicin biosynthesis	2	1	0.02	1.65	1.00	0.47	0.00
Fructose and mannose metabolism	20	1	0.20	0.69	1.00	1.00	0.00
Pentose phosphate pathway	22	1	0.22	0.65	1.00	1.00	0.00
Alanine, aspartate and glutamate metabolism	28	1	0.27	0.56	1.00	1.00	0.00
Glyoxylate and dicarboxylate metabolism	32	1	0.31	0.51	1.00	1.00	0.00
Cysteine and methionine metabolism	33	1	0.31	0.50	1.00	1.00	0.00
Glycine, serine and threonine metabolism	34	1	0.32	0.49	1.00	1.00	0.00
Arginine and proline metabolism	38	1	0.35	0.45	1.00	1.00	0.00
Valine, leucine and isoleucine degradation	40	1	0.37	0.43	1.00	1.00	0.00

Raw p, raw P-value; FDR, false discovery rate.

Supplementary Table 3. Pathway analysis of liver metabolites detected in control and carrot-fed lambs.

Pathway	Total	Expected	Hits	Raw p	log10(p)	Holm adjust	FDR	Impact
Fructose and mannose metabolism	20	0.33	3.00	0.00	2.42	0.32	0.32	0.22
Glycerolipid metabolism	16	0.26	2.00	0.03	1.56	1.00	0.96	0.33
Pentose and glucuronate interconversions	18	0.30	2.00	0.03	1.47	1.00	0.96	0.30
Glycolysis / Gluconeogenesis	26	0.43	2.00	0.07	1.17	1.00	1.00	0.00
Galactose metabolism	27	0.45	2.00	0.07	1.15	1.00	1.00	0.00
Glyoxylate and dicarboxylate metabolism	32	0.53	2.00	0.10	1.02	1.00	1.00	0.10
Amino sugar and nucleotide sugar metabolism	37	0.61	2.00	0.12	0.91	1.00	1.00	0.08
Ascorbate and aldarate metabolism	10	0.17	1.00	0.15	0.81	1.00	1.00	0.00
Citrate cycle (TCA cycle)	20	0.33	1.00	0.29	0.55	1.00	1.00	0.05
Pentose phosphate pathway	22	0.36	1.00	0.31	0.51	1.00	1.00	0.00
Pyruvate metabolism	22	0.36	1.00	0.31	0.51	1.00	1.00	0.00
Propanoate metabolism	23	0.38	1.00	0.32	0.49	1.00	1.00	0.00
Alanine, aspartate and glutamate metabolism	28	0.46	1.00	0.38	0.43	1.00	1.00	0.09
Glycine, serine and threonine metabolism	34	0.56	1.00	0.44	0.36	1.00	1.00	0.02

Raw p, raw P-value; FDR, false discovery rate P-value

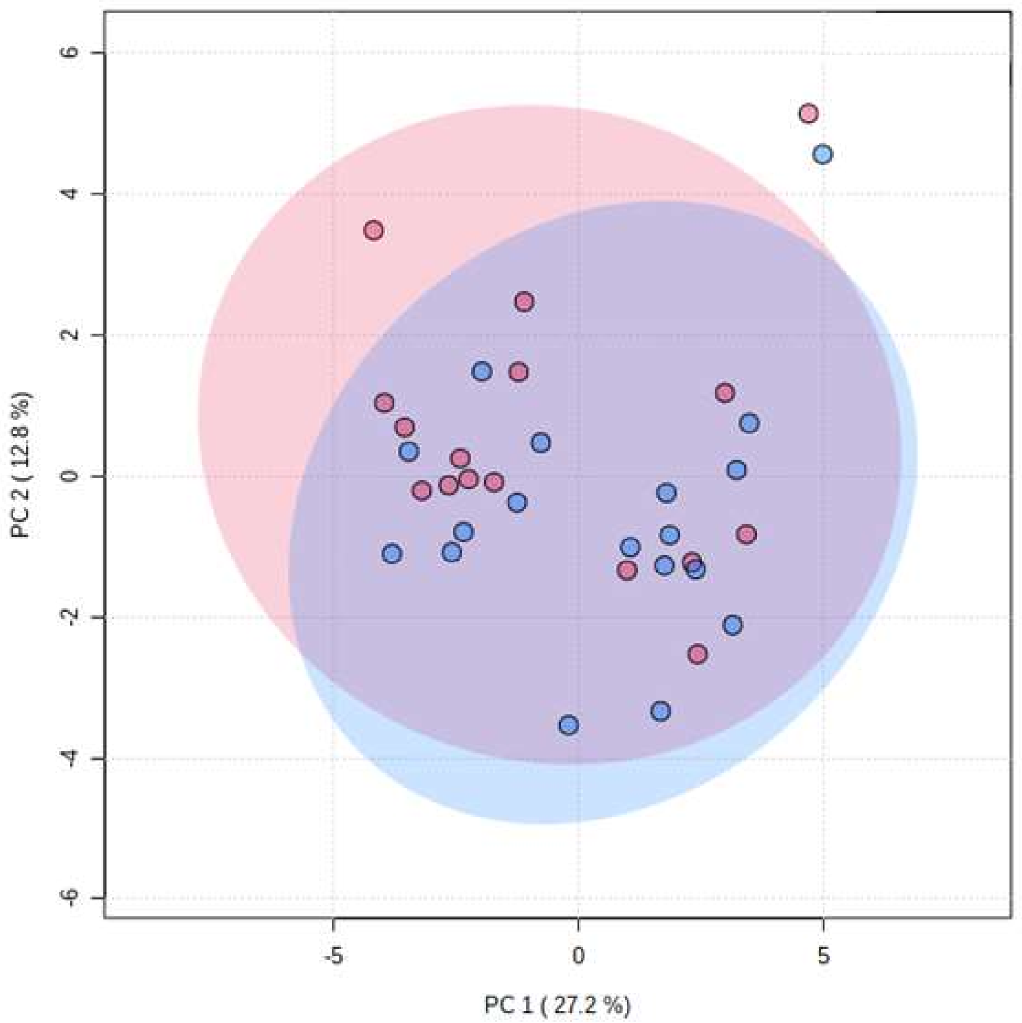

Supplementary Figure 2. Principal component analysis visualised via scores plot of plasma metabolic profiles obtained from the blood plasma of control- (red) or carrot-fed (blue) lambs. Of the 170 metabolites detected, none (FDR P-value > 0.05) were found to be influenced by diet.

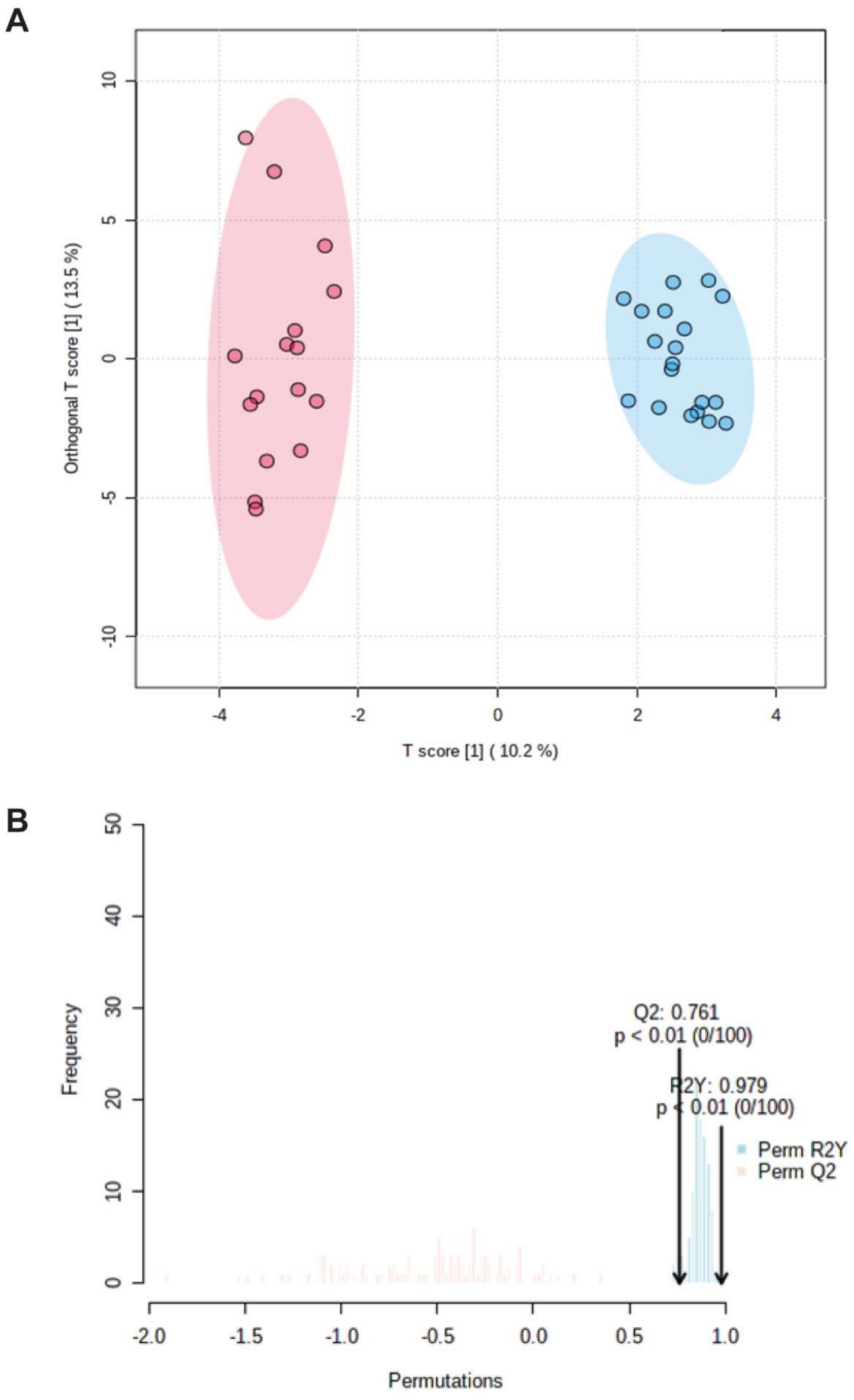

Supplementary Figure 3. (A) Orthogonal partial least squared discriminant analysis for metabolites identified on GC-MS to be differentially expressed, confirmed by t-test ($P < 0.05$). Each data point on (A) represents one lamb. (B) Validation of the rumen fluid OPLS-DA model after $n = 100$ permutations. Biological significance was determined with a Q^2 value > 0.5 and goodness of fit determined by R^2.
